# Heat Capacity and Thermodynamic Functions of Titanium-Manganites of Lanthanum, Lithium and Sodium of LaLi_2_TiMnO_6_ and LaNa_2_TiMnO_6_

**DOI:** 10.3390/molecules28135194

**Published:** 2023-07-04

**Authors:** Bulat Kunurovich Kasenov, Shuga Bulatovna Kasenova, Zhenisgul Imangalievna Sagintaeva, Sailaubai Baisanov, Natalya Yulievna Lu, Altynbek Nukhuly, Erbolat Ermekovich Kuanyshbekov

**Affiliations:** 1 Laboratory of Thermochemical Processes, Zh. Abishev Chemical-Metallurgical Institute, 100009 Karaganda, Kazakhstan; kasenovashuga@mail.ru (S.B.K.); kai_sagintaeva@mail.ru (Z.I.S.); mr.ero1986@mail.ru (E.E.K.); 2 Laboratory of Metallurgical Melts, Zh. Abishev Chemical-Metallurgical Institute, 100009 Karaganda, Kazakhstan; met.rasplav@mail.ru (S.B.); hmi_science@mail.ru (N.Y.L.); 3 Department of Chemistry, Pavlodar Pedagogical University, 140002 Pavlodar, Kazakhstan; nukhuly@mail.ru

**Keywords:** titanium-manganite, lithium, sodium, lanthanum, heat capacity, standard entropy, standard enthalpy of formation, phase transition, thermodynamic functions, electrophysics

## Abstract

Titanium-manganites of LaLi_2_TiMnO_6_ and LaNa_2_TiMnO_6_ were synthesized by the methods of ceramic technology from the oxides of lanthanum, titanium (IV), manganese (III), and the carbonates of lithium and sodium. The types of their syngony and the parameters of their gratings were determined radiographically. The isobaric heat capacities of titanium-manganites were measured with experimental calorimetry in the range of 298.15–673 K. It was found that on the dependence curve of heat capacity versus temperature of C°_p_~f(T), for LaLi_2_TiMnO_6_ at 348 K and 598 K, and LaNa_2_TiMnO_6_ at 348 K, there are abnormal jumps in heat capacity, probably related to phase transitions of the second kind. Taking into account the temperatures of the phase transitions, the equations of the temperature dependence of the heat capacity of titanium-manganites were derived. Their standard entropies were calculated by the ion increments method. Temperature dependences of the thermodynamic functions of S°(T), H°(T)-H°(298.15), and Φ^xx^(T) were calculated using the experimental data on heat capacities and the calculated values of the standard entropies. The standard heat capacities of the studied compounds were calculated by the independent methods of ion increments and Debye, the values of which were in satisfactory agreement with the experimental data. The standard enthalpy of the formation of LaLi_2_TiMnO_6_ and LaNa_2_TiMnO_6_ was calculated according to the methodology developed by the authors. The conducted electrophysical studies determined the nature of the second-order phase transition and the semiconductor features of their conductivity. Thus, all the above-mentioned data on the experimental and calculated studies of the temperature dependence of heat capacity, the thermodynamic functions to determine a standard enthalpy of formation of LaLi_2_TiMnO_6_ and LaNa_2_TiMnO_6_, and the investigation of their electrical properties are absolutely new, and they have no analogues.

## 1. Introduction

Ferroelectric materials are of interest in developing electrically controlled ultrahigh frequency devices [1]. They include manganites and compounds based on titanium dioxide and have unique physical and physical–chemical properties [2,3]. Manganites–perovskites, as representatives of strongly correlated systems, are currently the subject of intensive research; this is primarily due to the colossal magnetoresistance (CMS) observed in manganites. Such CMR values allow the use of manganites in the field of spin electronics: magnetic sensors, magnetoresistive reading heads, and magnetoresistive RAM [4]. Recently, manganites have been considered promising materials for creating magnetic refrigerators operating at room temperatures, which are compact, highly efficient, and environmentally safe [5].

It should be noted that the authors in [6] obtained substituted lanthanum–strontium manganites La_0.7_Sr_0.3_Mn_0.9_Me_0.1_O_3 ± δ_ (Me = Ti, Cr, Fe, Cu) using standard ceramic and glycerin-nitrate technologies. Their crystal structure was studied by high-temperature X-ray powder diffraction, thermal expansion coefficients were calculated, and electrical conductivity was investigated. In [7], the structural, electrical, and magnetic properties of the La_0.7_Sr_0.3_Mn_1-x_Ti_x_O_3_ system, which is characterized by rhombohedral distortion of the structure, were considered. It was found that the substitution of manganese ions with titanium ions leads to a weakening of ferromagnetism and an increase in resistivity. The study [8], presents the results of a study of the electrochemical properties of perovskite-like solid solutions (La_0.5+x_Sr_0.5−x_)_1–y_Mn_0.5_Ti_0.5_O_3−δ_ (x = 0–0.25, y = 0–0.03), synthesized by the citrate method, and studied as an oxide anode materials for solid oxide fuel cells. The authors in [9] present the structural, magnetic, and electrical properties of mixed Ti-MnSr_(1−x)_La_(x)_Ti_(0.5)_Mn_(0.5)_O_3_ (0 ≤ x ≤ 0.5). X-ray absorption spectroscopic measurements show that the addition of La^3+^ is compensated by a partial reduction of Mn^4+^ to Mn^3+^.

Along with manganites, semiconductor titanium oxides with transition metal impurities also attract attention as promising materials for their use in spin electronics and catalysis [10]. These include barium titanate, a traditional electro-ceramic material with properties of ferro-, ferroelectric, and paraelectric. It should be emphasized that the high values of the dielectric permittivity of ferroelectrics near the phase transition temperature allow them to be used in miniature capacitors [11]. The solid electrolytes of Li_0.35_La_0.55_TiO_3_-x wt.% LiF (LLTO–FX, x = 0.2, 4 and 6) were synthesized by the solid-phase reaction, as described in [12]; all samples formed a perovskite structure and the grain size gradually enlarged with increasing LiF content. The LLTO-F2 electrolyte showed high conductivity at a low activation energy of 0.26 eV; therefore, it is suitable to use in solid-state batteries. Titanates of Bi_2_Pr_2_Ti_3_O_12_ and Bi_2_Nd_2_Ti_3_O_12_ were obtained with solid-phase synthesis and sintered in air at temperatures of 1003–1323 K of the stoichiometric mixtures of Bi_2_O_3_, Nd_2_O_3_, Pr_6_O_11_, and TiO_2_. Their crystal structure was detected by X-ray diffraction. The high-temperature heat capacity was determined by differential scanning calorimetry. Based on the experimental data of C_p_ *=* f(T), the basic thermodynamic functions were calculated [13]. The heat capacities and thermodynamic characteristics of K_2_La_2_Ti_3_O_10_, K_2_Nd_2_Ti_3_O_10_, ErGaTi_2_O_7,_ DyGaTi_2_O_7_, and EuGaTi_2_O_7_ were studied by the experimental and calculated methods described in [14,15,16]. The research of the thermodynamic properties of double and ternary substituted manganites of the compositions of LaMe^I^Mn_2_O_5_, LnMe^II^Mn_2_O_5.5_, LnMe^I^_3_Mn_2_O_6_, and LnMe^II^_3_Mn_4_O_12_ (Me^I^-alkali, Me^II^-alkaline earth, Ln-rare-earth metals), was generalized in [17]. The above results demonstrate that the data available in the literature describe the thermodynamic properties of the individually substituted titanates and the individual manganites of the rare earth, alkali, and alkaline earth metals.

The study of the thermodynamic properties of substances is important for the directed synthesis of new compounds and materials. The thermodynamic functions in a wide temperature range are calculated during the study of heat capacity. Data on heat capacity allow the exploration of various ordering processes, determining the magnetic ferroelectric and superconductivity properties, etc. [18]. It should be stated that it is not possible to calculate the temperature dependences of enthalpy and entropy without heat capacity, i.e., the thermodynamic functions determining the direction of a chemical reaction such as Gibbs energy (ΔG) and the reduced thermodynamic potential (Φ**(T)) are calculated on their basis. However, the above data shows that there is no information in the literature about the synthesis and thermodynamic properties of combined double titanium-manganites of rare earth and alkali metals.

As a result of the above, the purpose of this study is a calorimetric investigation of heat capacity; calculations of the thermodynamic functions of the new titanium-manganites of LaLi_2_TiMnO_6_ and LaNa_2_TiMnO_6_; calculation of the fundamental thermodynamic constants of the studied compounds; the standard heat capacity, standard entropy, standard enthalpy of formation by the independent methods; and also the study of the temperature dependence of their electrophysical characteristics. This study is a continuation of our investigations; our results were summarized in [17]. The results obtained are of importance to predict the directed synthesis of the studied and analogous compounds, to analyze the heterogeneous equilibria according to II and III laws of thermodynamics involving titanium-manganites, and to discover their valuable physical and chemical properties. The new thermochemical constants of titanium-manganites are an initial data store to be included in fundamental reference books and information databanks.

## 2. Results and Discussion

Results of the calorimetric studies in Figure 5 and Table 7 describe that LaLi_2_TiMnO_6_ (at 348 K, 598 K) and LaNa_2_TiMnO_6_ (at 348 K) had anomalous discontinuities of heat capacity on the C°_p_~f(T) curve; this is probably related to the second-order phase transition. These transitions can be caused by Schottky effects, changes in the magnetic resistance, the electrical conductivity, the dielectric permittivity, Curie points, and Néel points, etc. [19]. Based on temperatures of the second-order phase transition, the equations of the temperature dependence of the heat capacity of titanium-manganites were calculated. They are described by the following equations (Table 1).

The resulting calculated curves and lines sufficiently describe the experimental data (Figure 5).

The graphs in Figure 5 are based on the experimental data and equations in Table 1, using the KOMPAS-3D LT software. For the reliability and correctness of the obtained straight lines and curves of the dependences of C°_p_~f(T), the calculated values of the heat capacities are also shown in Figure 5 between the experimental ones. Then, after the thermodynamic studies, we demonstrated the results of the electrophysical investigations to determine the nature of the mentioned second-order phase transitions.

In order to compare the phase transitions on the dependence curve C°_p_~f(T) of titanium-manganites, the temperature dependence of the heat capacity on the IT-S-400 of the standard substance of barium titanate (BaTiO_3_) was investigated. BaTiO_3_ (“p.a.”) corresponding to TU 6-09-3963-84 (purity of BaTiO_3_-99.8448%) was chosen to explore. It was analyzed by X-ray phase analysis using DRON-2.0 to compare with the reference data.

All the diffraction maxima on the X-ray photograph of BaTiO_3_ were equal to 4.04, 2.87, 2.35, 2.05, 1.83, and 1.66; 1.44 Å corresponded to data in the ASTM database [20].

**Figure 1:**
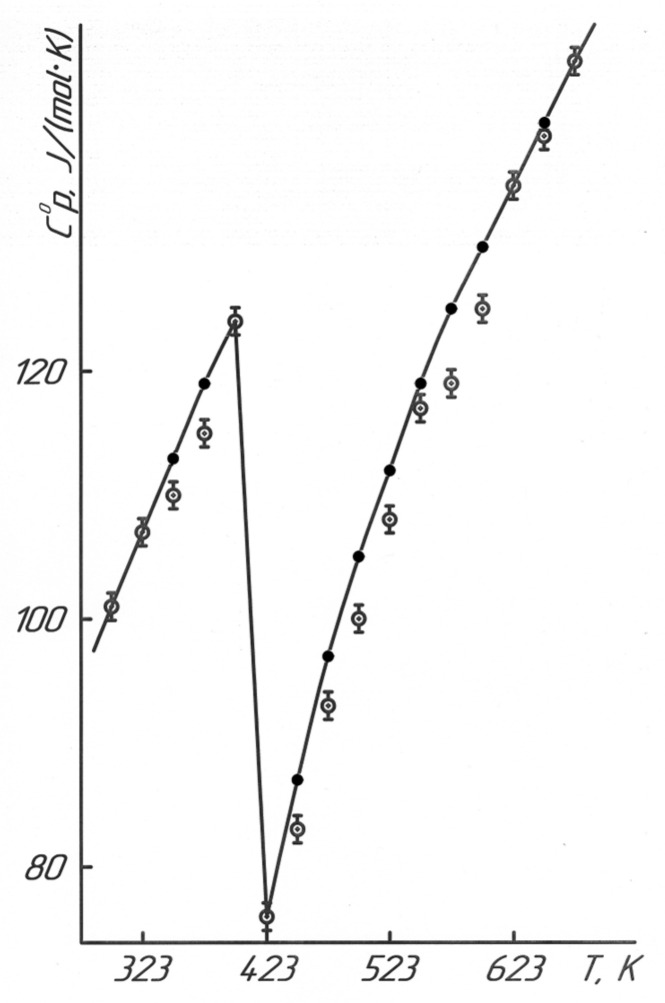
Temperature dependence of the heat capacity of BaTiO_3_. 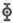
—experimental data, ●—calculated data.

Figure 1 and Table 2 show the research results of BaTiO_3_ heat capacity in the range of 298.15–673 K.

It should be stated that the experimental value of the standard heat capacity of BaTiO_3_ was equal to 101 ± 7 J/(mol·K), which is in good agreement with its reference data of 102.45 J/(mol·K), derived on the basis of the equation of the temperature dependence of the heat capacity (J/(mol·K)) [21]:

C°_p_ = 121.46 + 8.535 × 10^−3^T − 19.163 × 10^5^T^−2^ (298.15 − 1800 K). (1)

The experimental value of C°_p_(298.15) for BaTiO_3_ also corresponds well with its calculated value equal to 100.3 J/(mol·K). It was calculated by the method of the ionic entropic increments [2] under the formula:

S°(298.15) BaTiO_3_ = S^i^Ba^2+^ + S^i^TiO_3_^2−^, (2)

where S^i^Ba^2+^ and S^i^TiO_3_^2−^-ionic entropic increments equal 28.4 and 71.9 J/(mol·K), respectively [22].

Figure 1 illustrates the dependence diagram of C°_p_~f(T) for BaTiO_3_ in the interval of 298.15–673 K.

The data in Figure 1 and Table 2 demonstrate that the phase transition was observed in BaTiO_3_ at 398 K (125 °C). Referring to the literature data [23], this transition is observed at 393 K (120 °C) with a transition of its tetragonal modification to a cubic one with the appearance of the Curie point. The temperature dependence of the heat capacity of BaTiO_3_ was studied, and it depended on the heating rate of 1, 3, and 5 K/min, as described in [24]. This phase transition was observed at 395 K, 394.1 K, and 390.9 K, respectively. The technical capabilities of the IT-C-400 calorimeter can measure the heat capacities only per 25 K (in this interval of 373–398 K); thus, the temperature of this observed phase transition at 398 K is quite correct. Based on the temperature of the phase transition (398 K), we derived the equations describing this temperature dependence (J/(mol·K)):

C°_p(1)_ = (45.2 ± 2.5) + (207.8 ± 11.5) × 10^−3^T − (5.64 ± 0.31) × 10^5^T^−2^      (298.15–398 K), (3)

C°_p(2)_ = (885.6 ± 49.1) − (1912.8 ± 105.97) × 10^−3^T      (398–423 K), (4)

C°_p(3)_ = (137.5 ± 7.6) + (62.1 ± 3.4) × 10^−3^T − (156.22 ± 8.65) × 10^5^T^−2^      (423–673 K). (5)

The technical capabilities of the calorimeter made it possible to calculate the standard entropies of compounds by using a system of ionic entropy increments [22]. These were equal to 203 ± 6 and 244 ± 7 J/(mol^.^K), respectively, for LaLi_2_TiMnO_6_ and LaNa_2_TiMnO_6_.

**Figure 2:**
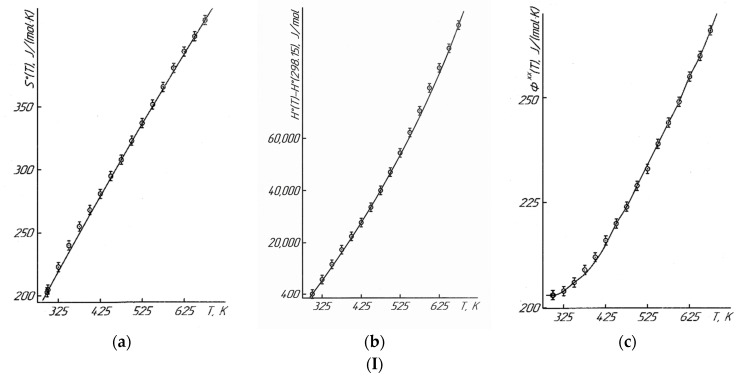

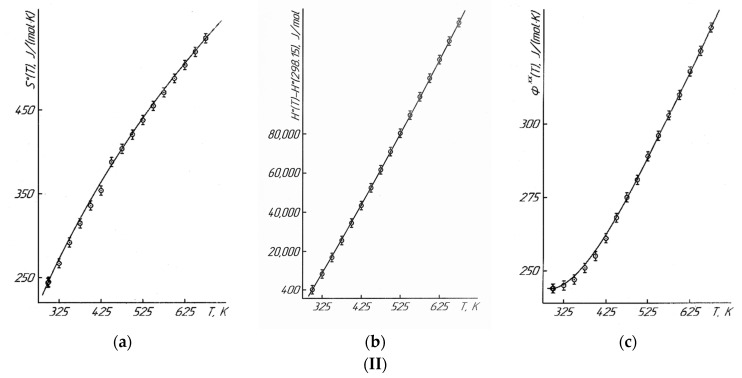
Dependence of functions of S°(T) (**a**), H°(T)-H°(298.15) (**b**), Φ^xx^(T) (**c**) of LaLi_2_TiMnO_6_ (**I**) and LaNa_2_TiMnO_6_ (**II**) on temperature. 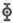
—experimental data.

The temperature dependences of C°_p_(T) and the thermodynamic functions of S°(T), H°(T)–H°(298.15), and Φ^xx^(T) (Table 3, Figure 2), in the interval of 298.15–675 K, were calculated by equations [25]:

(6) $$H{}^{\circ}(T)-H{}^{\circ}(298.15)=\int_{298.15}^{T}C_{p}^{0}dT,$$

(7) $$S{}^{\circ}(T)=S{}^{\circ}(298.15)+\int_{298.15}^{T}\frac{C_{p}}{T}dT,$$

(8) $$\Phi^{xx}(T)=S{}^{\circ}(T)-\frac{H{}^{\circ}(T)-H{}^{\circ}(298.15)}{T}$$

The temperature dependences of C°_p_(T) and the thermodynamic functions of S°(T), H°(T)–H°(298.15), and Φ^xx^(T) were calculated using the known ratios in the range of 298.15–675 K (Table 1). This temperature range was chosen using the fact that the Φ^xx^(T) function is only calculated from 298.15 K. It should be pointed out that the mentioned thermodynamic potential of Φ^xx^(T) is an important thermodynamic function necessary to calculate the chemical equilibria under the third law of thermodynamics. The errors of functions S°(T) and Φ^xx^(T) were calculated using the errors of S°(298.15) (±3.0%) [22] and the experimental data on C°_p_(T).

As a result, temperature dependences of the heat capacities of LaLi_2_TiMnO_6_ and LaNa_2_TiMnO_6_ in the range of 298.15–673 K were first studied.

To compare the values of the experimental data of the standard heat capacities of LaLi_2_TiMnO_6_ and LaNa_2_TiMnO_6_, they were calculated by independent calculation methods. According to [22], to calculate the C°_p_(298.15) of titanium-manganites, the following values of ion increments (C^i^_p_) of heat capacity [J/(mol·K] were used: Li^+^ = 20.7; Na^+^ = 26.8; La^3+^ = 29.3; Ti^4+^ = 25.5; Mn^3+^ = 25.0; O^2−^ = 16.7.

The calculation of the C°_p_(298.15) of titanium-manganites was carried out according to the scheme:

C°_p_(298.15)LaM^I^_2_TiMnO_6_ = C^i^_p_(298.15)La^3+^ + 2C^i^_p_(298.15)M^+^ + C^i^_p_(298.15)Ti^4+^ + C^i^_p_(298.15)Mn^3+^ + 6C^i^_p_(298.15)O^2−^, (9)

where C^i^_p_(298.15) is the increment of the ion heat capacity at 298.15. The values of C^o^_p_(298.15) were calculated according to Equation (9), and were equal, respectively, to 219.4 J/(mol·K) for LaLi_2_TiMnO_6_ and 231.6 J/(mol·K) for LaNa_2_TiMnO_6_, were in satisfactory agreement with their experimental values of 216.0 and 240.0 J/(mol·K).

The calculation of the standard heat capacity of LaNa_2_TiMnO_6_ by the Debye method [26] also gave good convergence with its experimental value. To calculate the C^o^_p_(298.15) LaNa_2_TiMnO_6_, the Debaev characteristic temperatures of the elements (*Q_D_*, K) that make up this titanium-manganite and their melting temperatures (*T_melt._*, K) were used. For *T_melt._* LaNa_2_TiMnO_6_, was 1473 K. The characteristic temperatures of the elements for LaNa_2_TiMnO_6_ (*Q_D_*) were determined by the Korefan equation [26]:

(10) $$Q_{D}^{\prime }=Q_{D}\cdot \sqrt{T_{melt}^{\prime }÷T_{melt}},$$

where $T_{\mathrm{melt.}}^{\prime }$ and *T_melt._* are the melting temperatures of the compound and element, respectively. Then we calculate the isochoric heat capacities of the elements using Debye functions, and by summing them, we found the isochoric heat capacity of LaNa_2_TiMnO_6_. The transition from isochoric heat capacity to isobaric was carried out according to the Nernst-Lindeman equation:

C_p_ = C_v_ + 0.0051·*T*·C_p_^2^/*T_melt._* (11)

Taking the above into account, the following data were used to calculate the standard heat capacity of LaNa_2_TiMnO_6_: *T_melt._*, K: La = 1193; Na = 370.7; Ti = 1941; Mn = 1517; O_2_ = 54.7; characteristic temperatures *Q_D_*, K: La = 135; Na = 370.7; Ti = 380; Mn = 303; O_2_ = 89 [26]. Using Equation (11), we calculated *Q_D_* for La = 150.01; Na = 308.97; Ti = 331.03; Mn = 298.57; O_2_ = 461.85. Then the arguments of the Debye function ($Q_{D}^{\prime }$/*T*) were calculated using tabular data in [26], equal to La = 0.503; Na = 1.036; Ti = 1.11; Mn = 1.001; O_2_ = 1.549. The corresponding isochoric heat capacities relative to *Q’_D_*/*T* based on tabular data [26] were equal for La = 24.64; Na = 23.75; Ti = 23.48; Mn = 23.76; O_2_ = 22.08 J/(mol·K).

Then by the equation:

C_v_ LaNa_2_TiMnO_6_ = C_v_ La + 2C_v_ Na + C_v_ Ti + C_v_ Mn + 3C_v_ O_2_, (12)

we calculated the isochoric heat capacity of LaNa_2_TiMnO_6_, equal to 185.62 J/(mol·K).

Further, according to the Nernst–Lindeman Equation (11), we calculated the standard isobaric heat capacity of C^o^_p_(298.15) LaNa_2_TiMnO_6_, equal to 250.3 J/(mol·K). This calculated value was in satisfactory agreement with the experimental value of the C^o^_p_(298.15) LaNa_2_TiMnO_6_ (240 ± 11 250.3 J/(mol·K)), with an accuracy of 4.1%. Thus, it shows the correctness of our experimental data. Therefore, values of the calculated standard heat capacity of titanium manganites calculated by the independent methods confirmed the correctness and reliability of their experimental values.

In order to determine the nature of the second-order phase transition on the curves of dependences (C°_p_~f(T)) of LaLi_2_TiMnO_6_ and LaNa_2_TiMnO_6_ in the interval of 293–483 K per 10 K step, their electrical properties were studied, as described in [27] on an LCR-781 serial device (Taiwan) operating at a frequency of 1 kHz. The accuracy of measurements of the electric capacity, relative dielectric permittivity (ε), and electrical resistivity (R), according to the datasheet, is ±0.05% [27]. The research technique was described in detail in [28] and in our similar study [29]. The dielectric permittivity of a standard substance of barium titanate (BaTiO_3_) was measured at 1 kHz to confirm the validity of the obtained data. We have described before the purity of the used BaTiO_3_ in our thermodynamic studies.

**Figure 3:**
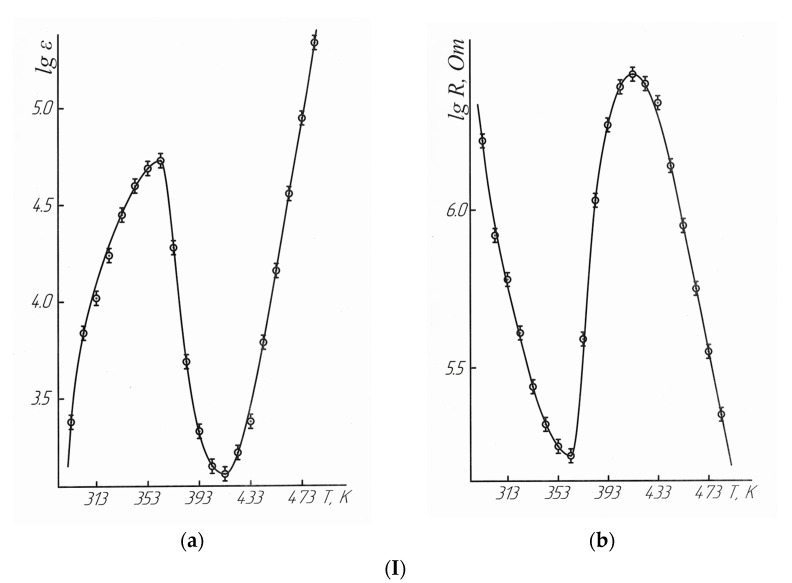

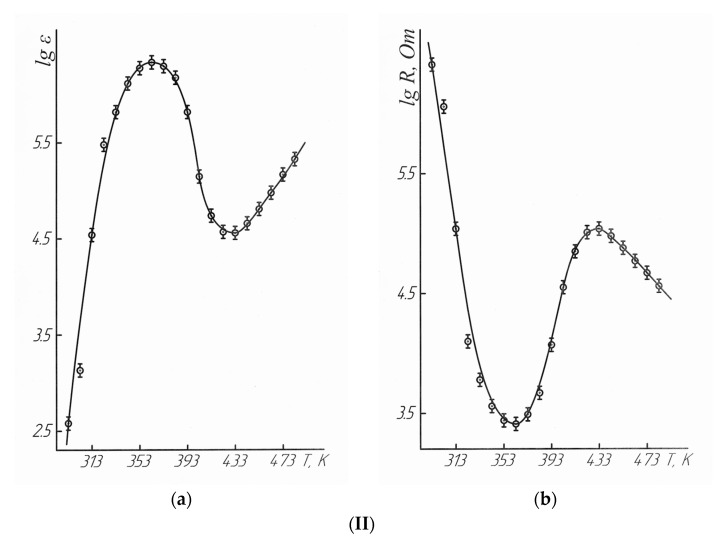
Dependence of the dielectric permittivity (**a**), electrical resistivity (**b**) of LaLi_2_TiMnO_6_ (**I**) and LaNa_2_TiMnO_6_ (**II**) on temperature. 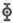
—experimental data.

The obtained value of the dielectric permittivity of BaTiO_3_ at 293 K is 1296, which conforms satisfactorily to its recommended value of 1400 ± 250 [30,31,32]. Table 4 and Figure 3 below demonstrate the results of the electrophysical measurements.

The data in Table 4 and Figure 3 demonstrate that LaLi_2_TiMnO_6_ in the range of 293–363 K had semiconductor conductivity. It had metallic conductivity at 363–413 K, and it had semiconductor conductivity again at 413–483 K. LaNa_2_TiMnO_6_ in the range of 293–363 K shows the semiconductor conductivity. Then, it had the metallic conductivity at 363–433 K, and the semiconductor conductivity was again observed at 433–483 K. The above-mentioned changes from the semiconductor to metallic conductivity indicate the nature of the second-order phase transition on the dependence curves of LaLi_2_TiMnO_6_ and LaNa_2_TiMnO_6_ at 348 K. It should also be stated that LaLi_2_TiMnO_6_ and LaNa_2_TiMnO_6_ at 363 K have the maximum values of the dielectric permittivity equal to 53,224 and 2,182,878 respectively and, thus, this also explains the nature of phase transitions.

These experiments demonstrated that the phase transitions of LaLi_2_TiMnO_6_ and LaNa_2_TiMnO_6_ on the dependence curves of thermal capacity versus temperature at 348 K correspond in the tested temperature range of 293–363 K (maximum at 363 K) to a transition from the semiconductor to metallic conductivity. They are characterized by the maximum values of the dielectric permittivity. The high values of the dielectric permittivity of ferroelectrics near a temperature of the phase transition are described in [11]. The activation energies of conductivity were calculated for LaLi_2_TiMnO_6_ (21.44 kJ/mol) and LaNa_2_TiMnO_6_ (75.49 kJ/mol).

The widths of band gaps for LaLi_2_TiMnO_6_ in the range of 293–363 K and 413–483 K were equal to 0.69 and 2.31 eV, respectively. The widths of band gaps for LaNa_2_TiMnO_6_ between 293–363 K and 433–483 K were equal to 1.02 and 1.83 eV. Thus, they can be classified as narrow-band semiconductors.

In order to calculate the values of the standard enthalpies of formation of the test titanium-manganites, our developed method was used to calculate the standard enthalpy of formation of the double and triple manganites of the rare earth, alkali, and alkaline earth metals of the composition of LnMeI_3_MeII_3_Mn_4_O_12_ (MeI—alkali, MeII—alkaline earth, Ln—rare earth metals) [33,34].

The calculation method is as follows: the similarity coefficient *K*_1_ was calculated from the ratio of

*K*_1_ = Δ_ƒ_H°(298.15)Ln(MnO_4_)_3_/Δ_ok_H°(298.15) Ln(MnO_4_)_3_, (13)

where Δ*_f_*H°(298.15)Ln(MnO_4_)_3_ is a standard enthalpy of formation of permanganate of the rare-earth metals from the simple substances, Δ_ok_H°(298.15)Ln(MnO_4_)_3_ is a sum of the enthalpy of formation from simple oxides or the conditionally taken standard enthalpy of formation of permanganate of the rare earth metals from oxides, and is equal to

Δ_ok_H°(298.15) Ln(MnO_4_)_3_ = 0.5Δ*_f_*H°(298.15) Ln_2_O_3_ + 1.5Δ_ƒ_H°(298.15) Mn_2_O_7_. (14)

Then the similarity coefficient *K*_2_ was calculated under the equation of

*K*_2_ = Δ*_f_*H°(298.15) MeIMnO_4_/Δ_ok_H°(298.15) MeIMnO_4_, (15)

where Δ_ok_H°(298.15)MeIMnO_4_—a standard enthalpy of formation of permanganate of alkali metal from oxides is equal to

Δ_ok_H°(298.15) MeIMnO_4_ = Δ*_f_*H°(298.15) Me_2_O + 0.5Δ*_f_*H°(298.15) Mn_2_O_7_. (16)

The similarity coefficient *K*_3_ was calculated from the ratio of

*K*_3_ = Δ*_f_*H°(298.15) MeII(MnO_4_)_2_/Δ_ok_H°(298.15) MeII(MnO_4_)_2_, (17)

where Δ_ok_H°(298.15)MeII(MnO_4_)_2_ is a standard enthalpy of formation of permanganate of the alkaline earth metal from oxides is equal to

Δ_ok_H°(298.15) MeII(MnO_4_)_2_ = Δ*_f_*H°(298.15) MeO + Δ*_f_*H°(298.15) Mn_2_O_7_. (18)

The average similarity coefficient $\overset{-}{K}$ was calculated from

(19) $$\overset{-}{K}=(K_{1}+K_{2}+K_{3})/3$$

Δ_ok_H°(298.15) LnMeI_3_MeII_3_Mn_4_O_12_ was calculated from:

Δ_ok_H°(298.15)LnMeI_3_MeII_3_Mn_4_O_12_ = 0.5Δ*_f_*H°(298.15)Ln_2_O_3_ + 1.5Δ*_f_*H°(298.15)Me_2_O + 3Δ*_f_*H°(298.15)MeO + 2Δ*_f_*H°(298.15)Mn_2_O_3_. (20)

Similar to Equations (13), (15) and (19), the ratio can be described as:

(21) $$\overset{-}{K}=\Delta_{f}H{}^{\circ}(298.15)\;{\mathrm{LnMeI}}_{3}{\mathrm{LnMeII}}_{3}{\mathrm{Mn}}_{4}O_{12}/\Delta_{\mathrm{ok}}H{}^{\circ}(298.15)\;{\mathrm{LnMeI}}_{3}{\mathrm{LnMeII}}_{3}{\mathrm{Mn}}_{4}O_{12},$$

where it can be calculated as

(22) $$\Delta_{f}H{}^{\circ}(298.15)\;{\mathrm{LnMeI}}_{3}{\mathrm{MeII}}_{3}{\mathrm{Mn}}_{4}O_{12}=\overset{-}{K}\Delta_{\mathrm{ok}}H{}^{\circ}(298.15)\;{\mathrm{LnMeI}}_{3}{\mathrm{MeII}}_{3}{\mathrm{Mn}}_{4}O_{12}$$

In connection with the absence of the reference date on Δ*_f_*H°(298.15) manganites, the first approximate values $\overset{-}{K}$ were calculated from data on Δ*_f_*H°(298.15) permanganates, which were used to calculate the Δ*_f_*H°(298.15) of the test compounds. Thus, it was taken into account that the values $\overset{-}{K}$ of manganites are not much different from permanganates $\overset{-}{K}$.

Based on the above and taking the ratios of (24, 25) for the titanium-manganites of lanthanum, the alkali, and alkaline earth metals, the following ratios can be demonstrated as:

Δ*_f_*H°(298.15) LnMe^I^_3_Me^II^_3_Mn_4_O_12_/Δ_ok_H°(298.15) LnMe^I^_3_Me^II^_3_Mn_4_O_12_
= Δ*_f_*H°(298.15) LaMe^I^_2_TiMnO_6_/Δ_ok_H°(298.15) LaMe^I^_2_TiMnO_6_, (23)

Table 5 below shows the initial data for calculating the standard enthalpies of titanium-manganites formation, which are borrowed from [17,29,30,31].

It should be noted that the $\overset{-}{K}$ coefficient for calculating Δ_ƒ_H°(298.15) LaLi_2_TiMnO_6_ will be equal to 1.2375, and for LaNa_2_TiMnO_6_ − 1.3084.

The calculated values based on the above data Δ*_f_*H°(298.15) will be equal to −3607.0 kJ/mol for LaLi_2_TiMnO_6_ and −3579.3 kJ/mol for LaNa_2_TiMnO_6_, respectively.

## 3. Experimental Part

Titanium-manganites of LaMe^I^_2_TiMnO_6_ (Me^I^–Li, Na) were obtained by high-temperature synthesis using ceramic technology. Oxides of lanthanum (III) (“puriss. spec.”), titanium (IV), manganese (III), and carbonates of lithium and sodium (“p.a.”) were applied to synthesize the titanium-manganites. These substances were pre-annealed at 300 °C to remove adsorption moisture. The calculated mole ratios of the starting reagents to obtain the final compound were thoroughly mixed and ground in an agate mortar. Synthesis was performed in stages in a SNOL laboratory furnace. The first stage was at 600 °C for 5 h, and the second step was to raise the temperature of the synthesis to 800 °C for 5 h. The third stage had a temperature at 1000 °C for 10 h and was repeated twice. The fourth stage was at 1200 °C for 4 h. After each temperature rise, the mixture was cooled down and milled. The final process had a low-temperature annealing at 400 °C for 10 h to obtain the low-temperature equilibrium phases. The formation of the equilibrium composition of the studied phases was monitored by X-ray diffraction analysis on the DRON-2.0 apparatus.

**Figure 4:**
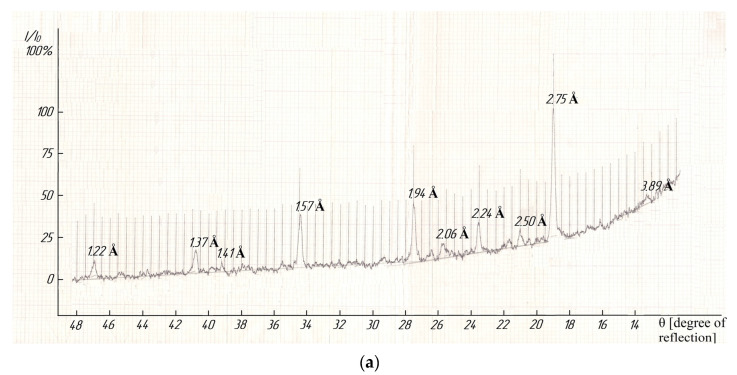

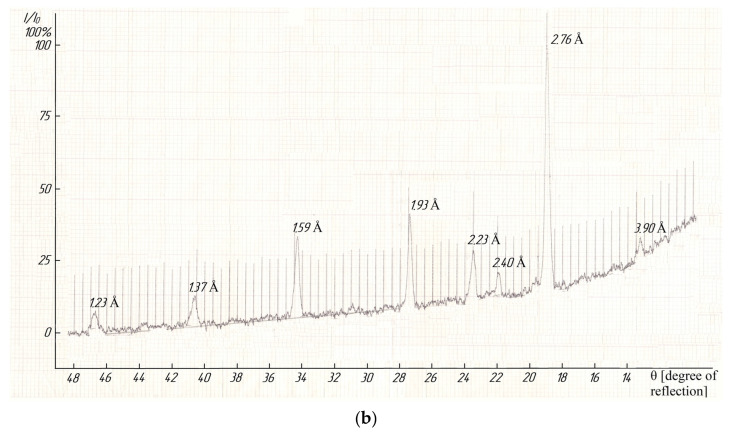
The X-ray photographs of LaLi_2_TiMnO_6_ (**a**) and LaNa_2_TiMnO_6_ (**b**).

The indexing of X-ray photographs demonstrated that compounds were crystallized in the cubic syngony with the lattice parameters as follows: LaLi_2_TiMnO_6_–a = 13.48 ± 0.02 Å, V° = 2449.46 ± 0.06 Å^3^, Z = 4, V^o^_el.cell_ = 612.87 ± 0.02 Å^3^, ρ_roent._ = 3.81; ρ_pick._ = 3.78 ± 0.03 g/cm^3^; LaNa_2_TiMnO_6_–a = 14.06 ± 0.02 Å, V° = 2779.43 ± 0.06 Å^3^, Z = 4, V°_el.cell_ = 694.96 ± 0.02 Å^3^, ρ_roent._ = 3.67; ρ_pick._ = 3.65 ± 0.01 g/cm^3^ [38]. A pycnometric density was determined by using toluene as an indifferent liquid according to a well-known method [39]. Figure 4 (below) illustrates the diffractograms of the studied titanium-manganites. It should be pointed out that we present in detail the results of the indexing of X-ray photographs of the above compounds, and their correctness and reliability were confirmed by good conformity between the experimental and calculated values of 10^4^/d^2^, and the pycnometric and X-ray densities of the theoretical and experimental values of their unit cells, as described in [38].

These compounds can be assigned to the perovskite structure according to the information below. The above-mentioned compounds can be represented as derivatives of lanthanum titanate (LaTiO_3_) and lanthanum manganate (LaMnO_3_), and they belong to a perovskite structure [40]. Secondly, in order to assign these compounds to the perovskite structure, we calculated the tolerance factor (*t*) by the formula [23]:

(24) $$t=\frac{\tau_{A}+\tau_{O}}{\sqrt{\tau_{B}}+\tau_{O}}$$

where $\tau_{A,}\tau_{B,}$ $\tau_{O}$–ion radii of A, B, and O^2^. In our case, A is a sum of ions of La^3+^, Li^+^, Ti^4+^ or La^3+^, Na^+^, Ti^4+^, and B-ion Mn^3+^. The radii of ions of La^3+^, Li^+^, Na^+^, Ti^4+^, Mn^3+^, and O^2−^ at a coordination number equal to 6 according to the Goldschmidt system were used, according to [41]. The Goldschmidt system was preferred, i.e., the Pauling system has no ion radius of Mn^3+^. The tolerance factors (*t*) for LaLi_2_TiMnO_6_ (*t* = 0.97) and LaNa_2_TiMnO_6_ (*t* = 0.94) were calculated by Equation (24). Additionally, we calculated the “tolerance factor” *t*, based on the system of ion radii according to Shannon and Previtt, for LaLi_2_TiMnO_6_ 0.94 and for LaNa_2_TiMnO_6_-0.90. The tolerance factor (*t*) is approximately in the range of 0.80 ÷ 1.00 for all perovskite-type compounds and *t* > 0.89 for an ideal cubic structure, as described in [41].

The parameter increment of “a” and the volume increase of the unit cells were observed during the elevating ionic radii from Li to Na.

Temperature dependence of the heat capacity of synthesized compounds of LaLi_2_TiMnO_6_ and LaNa_2_TiMnO_6_ was explored in the temperature range of 298.15–673 K by dynamic calorimetry using an IT-C-400 calorimeter. The device is based on the comparative method of a dynamic calorimeter with a heat meter and an adiabatic shell. During the experiment of heating (per 25 °C), the time lag of the ampoule temperature in relation to the base temperature was measured on an F136 device and stopwatch. Based on the specifications of the calorimeter, the heat capacity was measured per 25 K, and the limit temperature measurement was 673 K. The initial temperature for the measurement of heat capacity was 298.15 K and permitted to obtain the fundamental constant-standard heat capacity of the compound.

The measuring range of the volumetric heat capacity was not less than 1 × 10^6^ J/K·m^3^. The time for the full temperature range with the experimental data processing was no more than 2.5 h. The measurement errors on the IT-C-400 device did not exceed ±10%. The device was calibrated using a calculation of the thermal conductivity of the heat meter (*K_T_*) [42,43].

A value of the molar heat capacity was calculated from the specific heat capacity using the molar mass. At each temperature after 25 K, five parallel experiments were conducted, the results of which were averaged and processed by methods of mathematical statistics.

For the averaged values of the specific heat capacity at each temperature, the standard deviation ($\overset{-}{\delta }$) was estimated according to [43]:

(25) $$\delta =\sqrt{\frac{\sum_{i=1}^{n}{\left(C_{i}-\overset{-}{C}\right)}^{2}}{n-1}}$$

where *n*—number of experiments, *C_i_*—a measured value of specific heat capacity, and $\overset{-}{C}$—an arithmetic average of measured values of the specific heat capacity. A random error was calculated for averaged values of the molar heat capacity as described in [43]:

(26) $$\overset{\circ }{\Delta }=\frac{\delta \times t_{p}}{\overset{-}{C}}\times 100$$

where $\overset{\circ }{\Delta }$—a random error in % and *t_p_—*Student coefficient (for *n* = 5, *t_p_* = 2.75 at *p* = 0.95 of the confidence range).

Operation of the device was verified with a calculation of the heat capacity of α-Al_2_O_3_ (“p.a.”, TU 6.09-426-75). The repeated (parallel) measurements in the range of 173–673 K (at 25 K, 5 times) were performed for calibration and verification. Therefore, five parallel measurements were made at each temperature at 25 K. The results were averaged and processed using mathematical statistics. Liquid nitrogen served as the refrigerant.

Our results, with the new literature data in [44], were compared for the accuracy of the heat capacity measurements of α-Al_2_O_3_ (Table 6).

The data in Table 6 demonstrates that our results for the temperature dependence of the heat capacity of -Al_2_O_3_, in the range of 173–673 K, satisfactorily conformed to the results in [44] within the operating accuracy of the IT-C-400 calorimeter.

It should be stated that in order to compare our values of the heat capacities of Al_2_O_3_ with the data in [44], our experimental values were used at 10 and 50 K based on equations of C°_p_~f(T) calculated from the experimental data because the data in [44] for C°_p_~f(T) were used at 10 and 50 K, while our experimental data were measured at ΔT = 25 K. It should be noted that the real errors of the experimental data on heat capacities calculated with Equations (25) and (26) were lower than a limiting accuracy of the device, i.e., it was less than 10%.

**Figure 5:**
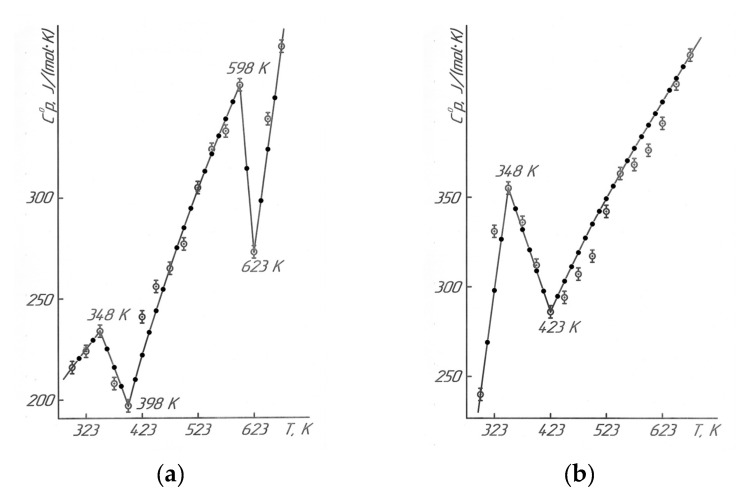
Temperature dependence of the heat capacity LaLi_2_TiMnO_6_ (**a**) and LaNa_2_TiMnO_6_ (**b**). 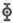
—experimental data, ●—calculated data.

Table 7 and Figure 5 demonstrate the results of the calorimetric studies.

## 4. Conclusions

The temperature dependences of the heat capacity of LaMeI_2_TiMnO_6_ (MeI–Li, Na) were studied for the first time by experimental dynamic calorimetry in the range of 298.15–673 K.

The anomalous discontinuities in heat capacity of LaLi_2_TiMnO_6_ (348 K, 598 K) and LaNa_2_TiMnO_6_ (348 K) were observed on C°_p_~f(T) curve. There were probably related to the second-order phase transitions.

Based on the temperatures of the phase transitions, the equations for the temperature dependence of heat capacity were derived, and they sufficiently describe the experimental data.

The temperature dependences of C°_p_(T) and the thermodynamic functions of S°(T), H°(T)-H°(298.15), and Φ^xx^(T) of the studied compounds were calculated using the experimental data on C°_p_(T) and the calculated values of S°(298.15) in the range of 298.15–673 K.

Using the methods of ion increments and Debye, the standard heat capacities of lithium and sodium lanthanum titanium-manganites were calculated, which were in satisfactory agreement with the experimental data.

According to the developed methodology, the standard enthalpy of the formation of LaLi_2_TiMnO_6_ and LaNa_2_TiMnO_6_ was calculated.

Based on the conducted electrophysical research, the nature of the second-order phase transitions on curves of C°_p_~f(T) dependences of the studied titanium-manganites was proved. Their semiconductor and metallic features of conductivity were revealed. The widths of their band gaps were calculated.

As a result, all the above-mentioned data on the experimental and calculated studies of the temperature dependence of heat capacity, thermodynamic functions, the results of calculation of the standard enthalpy of formation, and their revealed electrophysical characteristics of LaLi_2_TiMnO_6_ and LaNa_2_TiMnO_6_ are absolutely new, and they have no analogues.

The research results are also of practical importance for the directed synthesis of similar compounds with valuable physical and chemical properties, for prediction of the perspective physical and chemical characteristics of the studied phases, and for analysis of heterogeneous equilibria according to II and III laws of thermodynamics involving titanium-manganites. The new thermochemical constants are initial data to be loaded into fundamental guides for the thermodynamic values and information databanks. The conducted electrophysical studies determined the semiconducting character of conductivity of LaLi_2_TiMnO_6_ and LaNa_2_TiMnO_6_ and the maximum values of the dielectric permittivity near the temperature of the phase transitions.

## Figures and Tables

**Table 1:** Coefficients of equations for the temperature dependence of heat capacity.

Compound	Coefficients of Equation C°_p_ = a + b⋅T + c⋅T^−2^, J/(mol·K)			∆T, K
	a	b⋅10^−3^	c⋅10^5^	
LaLi_2_TiMnO_6_	107 ± 5	365.8 ± 17.0	-	298–348
	491 ± 23	−(737.3 ± 34.4)	-	348–398
	101 ± 5	502.4 ± 23.4	−(578.8 ± 7.7)	398–598
	2325 ± 108	−(3292.4 ± 153.4)	-	598–623
	−(988 ± 46)	2025.4 ± 94.4		623–673
LaNa_2_TiMnO_6_	−(444 ± 21)	2297.2 ± 106.1	-	298–348
	674 ± 31	−(916.9 ± 42.4)	-	348–423
	161 ± 7.45	431.0 ± 19.9	−(102.2 ± 4.7)	423–673

**Table 2:** The experimental values of BaTiO_3_ heat capacity [$C_{p}\;\pm \;\overset{-}{\delta }$, J/(g·K); C°_p_ ± $\overset{\circ }{\Delta }$, J/(mol·K)].

T, K	$C_{p}\;\pm \;\overset{-}{\delta }$	$C{}^{\circ}p_{\;}\pm \;\overset{\circ }{\Delta }$	T, K	$C_{p}\;\pm \;\overset{-}{\delta }$	$C{{{}^{\circ}}_{p}}^{\;}\pm \;\overset{\circ }{\Delta }$
298	0.4319 ± 0.0114	101 ± 7	498	0.4331 ± 0.0036	101 ± 3
323	0.4583 ± 0.0120	107 ± 8	523	0.4669 ± 0.1229	109 ± 4
348	0.47083 ± 0.0105	110 ± 7	548	0.5041 ± 0.0087	118 ± 8
373	0.49223 ± 0.0102	115 ± 7	573	0.5110 ± 0.0069	119 ± 9
398	0.5330 ± 0.0049	124 ± 3	598	0.5382 ± 0.0107	126 ± 7
423	0.3279 ± 0.0083	76 ± 5	623	0.5801 ± 0.0112	135 ± 8
448	0.3563 ± 0.0084	83 ± 5	648	0.6000 ± 0.0134	140 ± 7
473	0.3992 ± 0.0084	93 ± 4	673	0.6210 ± 0.0095	145 ± 6

**Table 3:** The thermodynamic functions of the compounds.

T, K	$C{{}^{\circ}}_{p}(T)\;\pm \;\overset{\circ }{\Delta }$ J/(mol·K)	$S{}^{\circ}(T)\;\pm \;\overset{\circ }{\Delta }$ J/(mol·K)	$H{}^{\circ}(T)–H{}^{\circ}(298.15)\;\pm \;\overset{\circ }{\Delta }$ J/mol	$\Phi^{\mathrm{xx}}(T)\;\pm \;\overset{\circ }{\Delta }$ J/(mol·K)
LaLi_2_TiMnO_6_				
298.15	216 ± 10	203 ± 6	-	203 ± 15
300	217 ± 10	205 ± 16	433 ± 20	203 ± 15
325	226 ± 11	223 ± 17	5960 ± 280	204 ± 16
350	235 ± 11	240 ± 18	11,720 ± 5160	206 ± 16
375	214 ± 10	255 ± 20	17,310 ± 800	209 ± 16
400	196 ± 9	268 ± 21	22,440 ± 1050	212 ± 16
425	224 ± 10	281 ± 22	27,730 ± 1290	216 ± 17
450	246 ± 11	295 ± 23	33,610 ± 1570	220 ± 17
475	267 ± 12	308 ± 24	40,030 ± 1860	224 ± 17
500	287 ± 13	323 ± 25	46,950 ± 2190	229 ± 18
525	305 ± 14	337 ± 26	54,350 ± 2530	233 ± 18
550	323 ± 15	352 ± 27	62,210 ± 2900	239 ± 19
575	340 ± 16	366 ± 28	70,510 ± 3290	244 ± 19
600	357 ± 17	381 ± 29	79,230 ± 3690	249 ± 19
625	267 ± 12	394 ± 30	86,930 ± 4050	255 ± 20
650	328 ± 15	406 ± 31	94,500 ± 4400	260 ± 20
675	379 ± 18	419 ± 32	103,340 ± 4820	266 ± 20
LaNa_2_TiMnO_6_				
298.15	240 ± 11	244 ± 7	-	244 ± 19
300	245 ± 11	245 ± 19	480 ± 20	244 ± 19
325	302 ± 14	267 ± 20	7320 ± 340	245 ± 19
350	360 ± 17	292 ± 22	15,600 ± 720	247 ± 19
375	330 ± 15	315 ± 24	24,140 ± 1110	251 ± 19
400	307 ± 14	336 ± 26	32,120 ± 1480	255 ± 19
425	285 ± 13	354 ± 27	39,520 ± 1830	261 ± 20
450	305 ± 14	388 ± 30	53,900 ± 2490	268 ± 20
475	321 ± 15	404 ± 31	61,710 ± 2850	275 ± 21
500	336 ± 16	421 ± 32	69,920 ± 3230	281 ± 21
525	350 ± 16	438 ± 33	78,500 ± 3500	289 ± 22
550	364 ± 17	455 ± 35	87,430 ± 4040	296 ± 23
575	378 ± 17	471 ± 36	96,710 ± 4470	303 ± 23
600	391 ± 18	488 ± 37	106,330 ± 4910	310 ± 24
625	404 ± 19	504 ± 38	116,280 ± 5370	318 ± 24
650	417 ± 19	520 ± 40	126,550 ± 5850	325 ± 25
675	430 ± 20	536 ± 41	137,130 ± 6340	333 ± 25

**Table 4:** Results of the electrophysical measurements.

T, K	R, Om	ε	lgε	lgR	R, Om	ε	lgε	lgR
LaLi_2_TiMnO_6_					LaNa_2_TiMnO_6_			
293	1,643,000	2381	3.38	6.22	2,549,000	379	2.58	6.41
303	832,700	6869	3.84	5.92	1,142,000	1351	3.13	6.06
313	604,400	10,571	4.02	5.78	109,000	34,515	4.54	5.04
323	404,300	17,528	4.24	5.61	12,560	305,305	5.48	4.10
333	272,600	28,456	4.45	5.44	6040	665,659	5.82	3.78
343	209,400	39,654	4.60	5.32	3636	1,308,086	6.12	3.56
353	177,400	48,911	4.69	5.25	2741	1,896,966	6.28	3.44
363	165,500	53,224	4.73	5.22	2580	2,182,878	6.34	3.41
373	392,800	19,155	4.28	5.59	3075	1,972,968	6.30	3.49
383	1,079,000	4849	3.69	6.03	4673	1,507,719	6.18	3.67
393	1,867,000	2119	3.33	6.27	11,750	653,784	5.82	4.07
403	2,428,000	1404	3.15	6.39	35,800	141,285	5.15	4.55
413	2,690,000	1294	3.11	6.43	70,030	55,338	4.74	4.85
423	2,510,000	1649	3.22	6.40	101,900	36,877	4.57	5.01
433	2,200,000	2379	3.38	6.34	109,100	36,200	4.56	5.04
443	1,371,000	6162	3.79	6.14	95,920	45,756	4.66	4.98
453	883,800	14,420	4.16	5.95	76,300	64,238	4.81	4.88
463	557,400	36,387	4.56	5.75	59,120	94,570	4.98	4.77
473	351,100	89,319	4.95	5.55	46,470	148,444	5.17	4.67
483	225,400	218,458	5.34	5.35	36,220	216,081	5.33	4.56

**Table 5:** Initial data for the calculation of standard enthalpy of titanium-manganites formation.

Compounds	−Δ_ok_H°(298.15)/ kJ/mol	$\overset{-}{K}$	−Δ*_f_*H°(298.15)/ kJ/mol	References
LaLi_3_Mg_3_Mn_4_O_12_	5514.4	1.2375	6824.1	[17]
LaNa_3_Ca_3_Mn_4_O_12_	5340.5	1.3084	6987.5	-//-
Li_2_O			593.94	[35]
Na_2_O			414.84	[35]
La_2_O_3_			1794.94	[36]
Mn_2_O_3_			957.72	[37]
TiO_2_			944.5	[37]

**Table 6:** Comparison of the heat capacity of α-Al_2_O_3_ with the literature data in [44] to verify the calorimeter operation.

T, K	C°_p_ (T), J/(mol·K)	
	Our Data	Data in [44]
180	44.50	43.83
230	64.86	61.18
250	70.37	67.08
280	77.07	74.82
300	76.31	79.41
350	86.49	88.86
400	94.12	95.21
450	100.26	101.8
500	105.47	106.1
550	110.09	109.7
600	114.29	112.5
650	118.20	114.9

**Table 7:** The experimental values of the heat capacity for the compounds.

T, K	$Cp_{\;}\pm \;\overset{-}{\delta }$, J/(g·K)	$C{{}^{\circ}}_{p}\;\pm \;\overset{\circ }{\Delta }$, J/(mol·K)	$Cp_{\;}\pm \;\overset{-}{\delta }$, J/(g·K)	$C{{}^{\circ}}_{p}\;\pm \;\overset{\circ }{\Delta }$, J/(mol·K)
	LaLi_2_TiMnO_6_		LaNa_2_TiMnO_6_	
298.15	0.6142 ± 0.0147	216 ± 14	0.6262 ± 0.0089	240 ± 11
323	0.6374 ± 0.0083	224 ± 8	0.8620 ± 0.0188	331 ± 15
348	0.6662 ± 0.0100	234 ± 10	0.9255 ± 0.0183	355 ± 16
373	0.5910 ± 0.0123	208 ± 12	0.8763 ± 0.0153	336 ± 16
398	0.5613 ± 0.0100	197 ± 10	0.8140 ± 0.0147	312 ± 14
423	0.6857 ± 0.0115	241 ± 11	0.7463 ± 0.0129	286 ± 13
448	0.7275 ± 0.0124	256 ± 12	0.7676 ± 0.0072	294 ± 14
473	0.7548 ± 0.0155	265 ± 15	0.8005 ± 0.0127	307 ± 14
498	0.7891 ± 0.0126	277 ± 12	0.8261 ± 0.0171	317 ± 15
523	0.8688 ± 0.0181	305 ± 18	0.8919 ± 0.0151	342 ± 16
548	0.9214 ± 0.0113	324 ± 11	0.9468 ± 0.0190	363 ± 17
573	0.9458 ± 0.0157	333 ± 15	0.9588 ± 0.0147	368 ± 17
598	1.0118 ± 0.0128	356 ± 13	0.9805 ± 0.0158	376 ± 17
623	0.7777 ± 0.0095	273 ± 9	1.0192 ± 0.0174	391 ± 18
648	0.9653 ± 0.0179	339 ± 17	1.0777 ± 0.0128	413 ± 19
673	1.0657 ± 0.0150	375 ± 15	1.1171 ± 0.0151	429 ± 20

## Data Availability

Not applicable.

## Sample Availability

Samples of the compounds are not available from the authors.
